# Biomedical generative pre-trained based transformer language model for age-related disease target discovery

**DOI:** 10.18632/aging.205055

**Published:** 2023-09-22

**Authors:** Diana Zagirova, Stefan Pushkov, Geoffrey Ho Duen Leung, Bonnie Hei Man Liu, Anatoly Urban, Denis Sidorenko, Aleksandr Kalashnikov, Ekaterina Kozlova, Vladimir Naumov, Frank W. Pun, Ivan V. Ozerov, Alex Aliper, Alex Zhavoronkov

**Affiliations:** 1 Insilico Medicine Hong Kong Ltd., Hong Kong Science and Technology Park, New Territories, Hong Kong, China; 2 Insilico Medicine AI Limited, Level 6, Unit 08, Block A, IRENA HQ Building, Masdar City, Abu Dhabi, UAE

**Keywords:** transformers, deep learning, therapeutic target discovery, aging biomarkers, human aging

## Abstract

Target discovery is crucial for the development of innovative therapeutics and diagnostics. However, current approaches often face limitations in efficiency, specificity, and scalability, necessitating the exploration of novel strategies for identifying and validating disease-relevant targets. Advances in natural language processing have provided new avenues for predicting potential therapeutic targets for various diseases. Here, we present a novel approach for predicting therapeutic targets using a large language model (LLM). We trained a domain-specific BioGPT model on a large corpus of biomedical literature consisting of grant text and developed a pipeline for generating target prediction. Our study demonstrates that pre-training of the LLM model with task-specific texts improves its performance. Applying the developed pipeline, we retrieved prospective aging and age-related disease targets and showed that these proteins are in correspondence with the database data. Moreover, we propose CCR5 and PTH as potential novel dual-purpose anti-aging and disease targets which were not previously identified as age-related but were highly ranked in our approach. Overall, our work highlights the high potential of transformer models in novel target prediction and provides a roadmap for future integration of AI approaches for addressing the intricate challenges presented in the biomedical field.

## INTRODUCTION

Aging, an inherent biological process, is characterized by the gradual decline in the efficiency of physiological and cellular functions over time, eventually leading to an increased susceptibility to diseases, dysfunction, and mortality [1]. Numerous studies have contributed to the current understanding of the interconnected events that occur at the molecular level concerning aging and longevity. Several directions have been extensively explored in this area from investigating telomere attrition, dysfunctional signaling pathways related to proteostasis, mTORC1 resistance, cellular and genomic instability [2] to identifying biomarkers, genetic variations, and the impact of distinct cell and tissue senescence on the age-related changes [3]. However, despite the current efforts of academia to dissect the molecular mechanisms underlying such processes, molecular mechanisms associated with aging remain not well-understood [4]. Aging, as a highly complex and multifaceted biological process, poses considerable challenges to traditional experimental and analytical methodologies in comprehensively deciphering its underlying mechanisms. Given the extensive interrelated network of genes, proteins, and pathways implicated in aging, unraveling such intricate associations demands a powerful approach capable of recognizing complex patterns.

The application of Artificial Intelligence (AI) has demonstrated promising success in numerous areas of biomedical research [5]. AI algorithms in drug discovery have revolutionized the field by significantly reducing the time and resources required to identify and develop new therapeutic compounds [6–8]. Recent advances in AI have expanded its use in aging research, a field that investigates the complex multifaceted mechanisms associated with the aging process [9]. Particularly, AI algorithms were successfully applied in the establishment of deep aging clocks that incorporate deep learning techniques to analyze a wide range of molecular and physiological changes that occur during the aging process [10–12]. Furthermore, AI has been successfully applied to predict age-related biological markers which are critical in the development of novel interventions to delay or reverse aging and its associated conditions [13–15]. This growing body of research highlights the transformative potential of AI and LLMs in the realm of aging and biomedical research, offering promising perspectives in the development of novel strategies to promote healthy aging and combat age-related diseases [6].

One of the key developments in AI is the emergence of large-scale language models (LLMs), which are powerful machine learning algorithms trained on vast amounts of text data to understand and generate language. These LLMs have been widely employed in diverse scientific disciplines, including biology and genomics, to enable sophisticated text-based analyses and predictions [16, 17]. Although the use of LLMs in the biomedical field still has not been comprehensively shown, we hypothesized that laden with the capacity to recognize intricate dependencies contained in vast volumes of scientific text, LLMs could present a powerful technique for age-centric research.

One of the important challenges for LLMs’ efficient usage is incipient approaches for the information retrieval from LLMs. Furthermore, since LLMs suffer from interpretability issues, it has been challenging to translate their predictive power into biologically meaningful insights [18]. Therefore, it is crucial to develop new methods that can effectively address the challenges associated with information extraction from LLMs and contribute to the understanding of complex biological networks and processes. Considering the limitations of the information retrieval from LLMs, we aimed to tackle this task in the application to target discovery within the framework of aging. We first devised a pipeline for information extraction from LLMs based on the estimation of the probability of the next token given the prompt. We applied this pipeline for the ranking of proteins according to their relationships with 14 age-related diseases and aging. Besides the task of target discovery as in our case, the proposed method could provide the basis in any task implying the ranking of the given list of subjects. We also showed that LLM pre-training with task-specific texts significantly impacts the overall model performance. Finally, we demonstrate the LLM-based approach is powerful in the identification of novel age-related targets, which were not previously reported by the standard target discovery techniques. Ultimately, our findings could provide the solid basis for further study of the efficient usage of LLMs in the domain-specific tasks. The presented pipeline proposed a new approach for target discovery and provides new insights into complex molecular mechanisms underlying pathologies and potential therapeutic strategies. Overall, our findings emphasize the importance of adopting data-driven approaches for identifying potential therapeutic targets and provide a roadmap for future research in the field.

## RESULTS

### The next token retrieval allows efficient information extraction from LLM

LLMs hold significant potential for biologically-related applications, although efficient extraction of the relevant information is a known obstacle for extended application of the models [19]. Most of the LLMs published recently are trained on the continuation of the text when the next word is chosen based on the context-aware words’ probability distribution learned from previously shown context [20]. Thus, the prediction of the next word given the prompt could be the promising methods for LLM-based information extraction in the task of ranging the list of known subjects. A medically important task with a matching objective is the target prioritization for a given disease. In this work, we proposed a pipeline for target discovery based on the LLM-retrieved probability of the gene to be the next word in the disease-related context.

**Figure 1:**
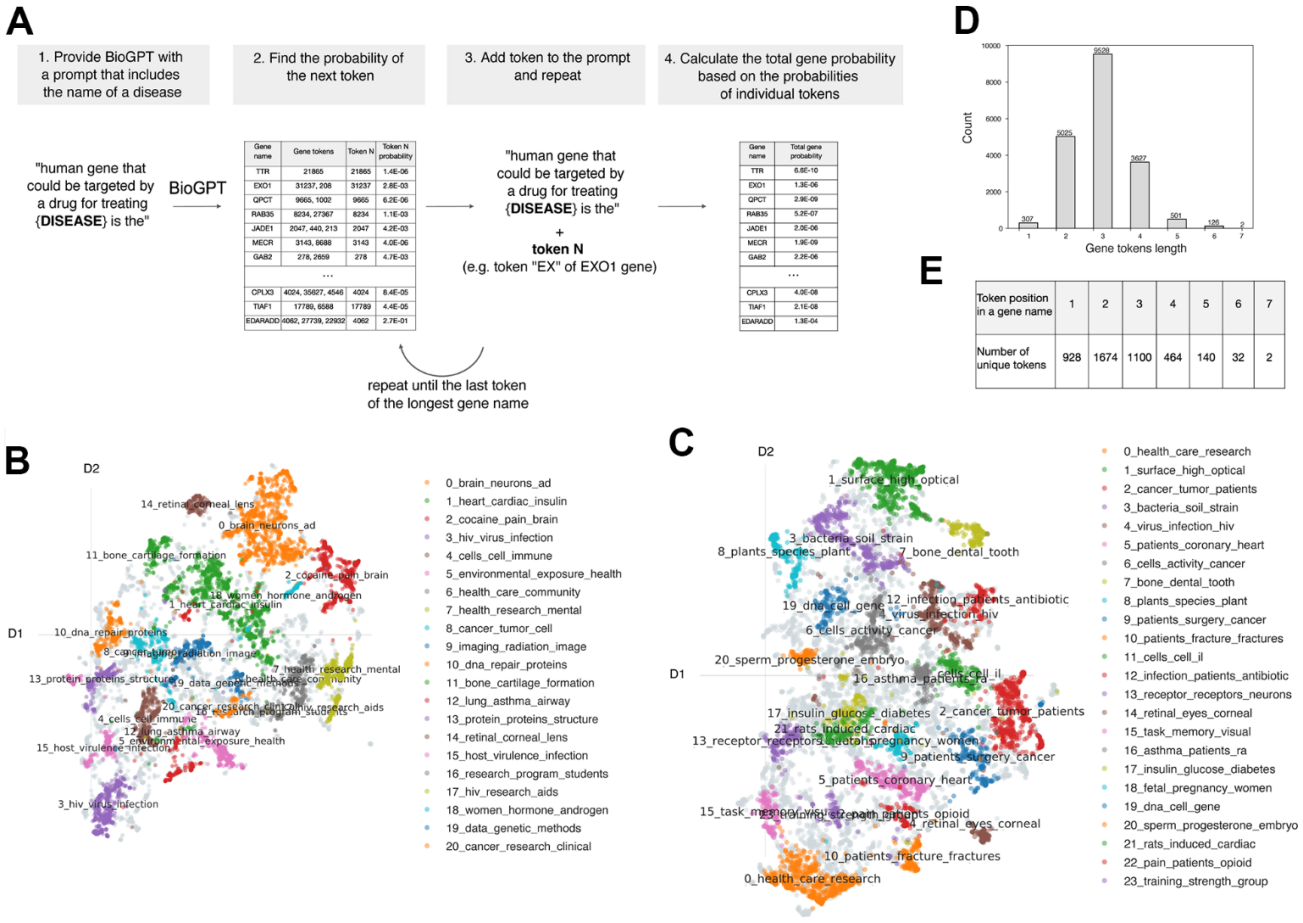
**The main method utilized in the work.** (**A**) The general pipeline of the work. (**B**) Predominant topics for the grant and (**C**) PubMed texts identified by BertTopic. (**D**) Distribution of token lengths for protein-coding genes. (**E**) The number of unique tokens placed in the noted positions within the gene name.

The general pipeline included the construction of the disease-related prompt, retrieval probability of several tokens continued from the initial prompt and the calculation of genes probability (Figure 1A). The main model used for the study was the BioGPT-based model that comprises the basic BioGPT with additional pre-training with more than 900K grants. BioGPT is a pre-trained transformer specifically developed for biomedical text generation and mining [21]. We hypothesized that additional training of the model with subject-related data distinguishable from the initial training data content allows the increase of model performance. Initial BioGPT was trained on the PubMed full text papers and abstracts that comprise articles, which are related to various domains of Life Sciences and not Biology specifically. Therefore, we intended to train the model with the data more relevant to target discovery for its further usage in target prediction tasks. We selected the internal InsilicoMedicine comprehensive dataset of grant proposals as they often contain detailed description of target mechanisms of action in the specific disease in contrast to PubMed paper that comprise general information along with pharmaceutically related texts.

We utilized the BERTopic model [22] for the identification of the prevalent topic within PubMed and grant texts. First, the results have indicated that topics are indeed different between these two text corpus. Moreover, the topics identified for grant proposals have appeared to be more relevant for target discovery (Figure 1B, 1C). The 20 main topics identified for grants were closely related to human health and specific conditions or organs (topics 0-4, 8, 10-12, 14-15, 17, 18) or health in general (topics 5 - 7, 9, 16, 19, 20). In contrast, topics of PubMed text were more heterogeneous. Also, along with the human-related information, there were articles devoted to animal studies (topics 3, 8, 21) and other fields of Life Science (topics 1). Altogether, training on grants, which is both distinct from the initial data corpus and contains target-related information, could be the promising approach for the model performance in the target discovery task.

We next optimized the prompt to make it suitable for the selected task of the next word prediction. Prompts were constructed in a way to increase the probability of the next word to be the abbreviation of the human gene. The efficiency of the prompt was estimated based on the number of genes found in the top 1K words ranged on the probability. The final prompt used for the task is as the following: “human gene targeted by a drug for treating {DISEASE} is the”. Notably, the increase in the prompt length negatively influenced the outcome while the addition of an article (i.e. “the” or “a”) at the end of the prompt had the opposite effect.

**Figure 2:**
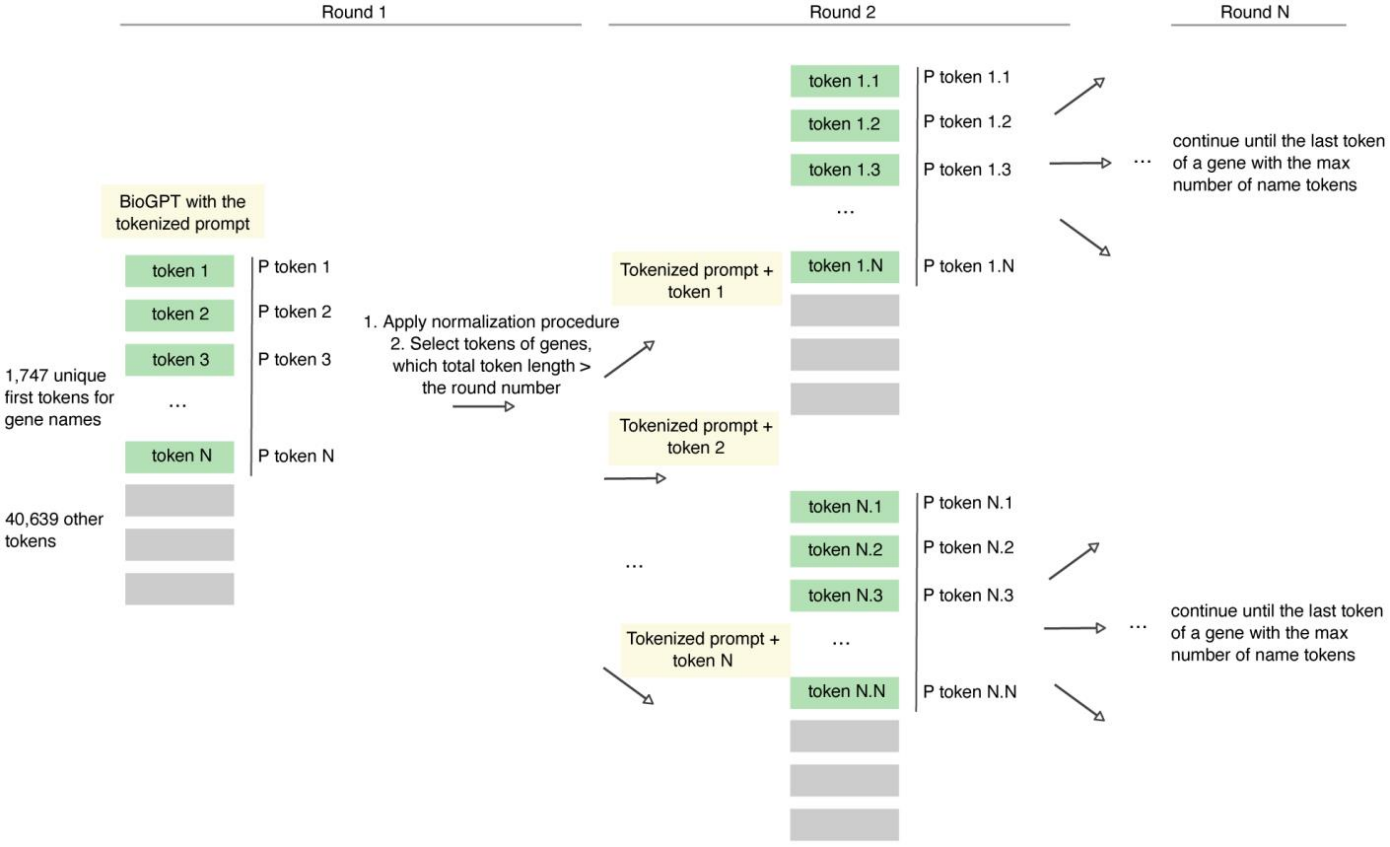
Overview of the established approach of the retrieval of the next token probability using BioGPT model.

One of the major parts of the pipeline development was to establish the procedure for the gene name probability estimation. We utilized the general tokenizer provided by Microsoft for BioGPT that contains only a part of the gene's names in the vocabulary. Thus, most of the genes are coded with 2 to 7 tokens, while only less than 2% of the genes are directly found in the vocabulary (Figure 1D, 1E). Thus, to estimate the probability of the gene name with several tokens, the tokens were iteratively added to the initial prompt for the calculation of the next token probability (Figure 2). The total probability of the gene was calculated as the multiplication of its tokens probabilities.

**Figure 3:**
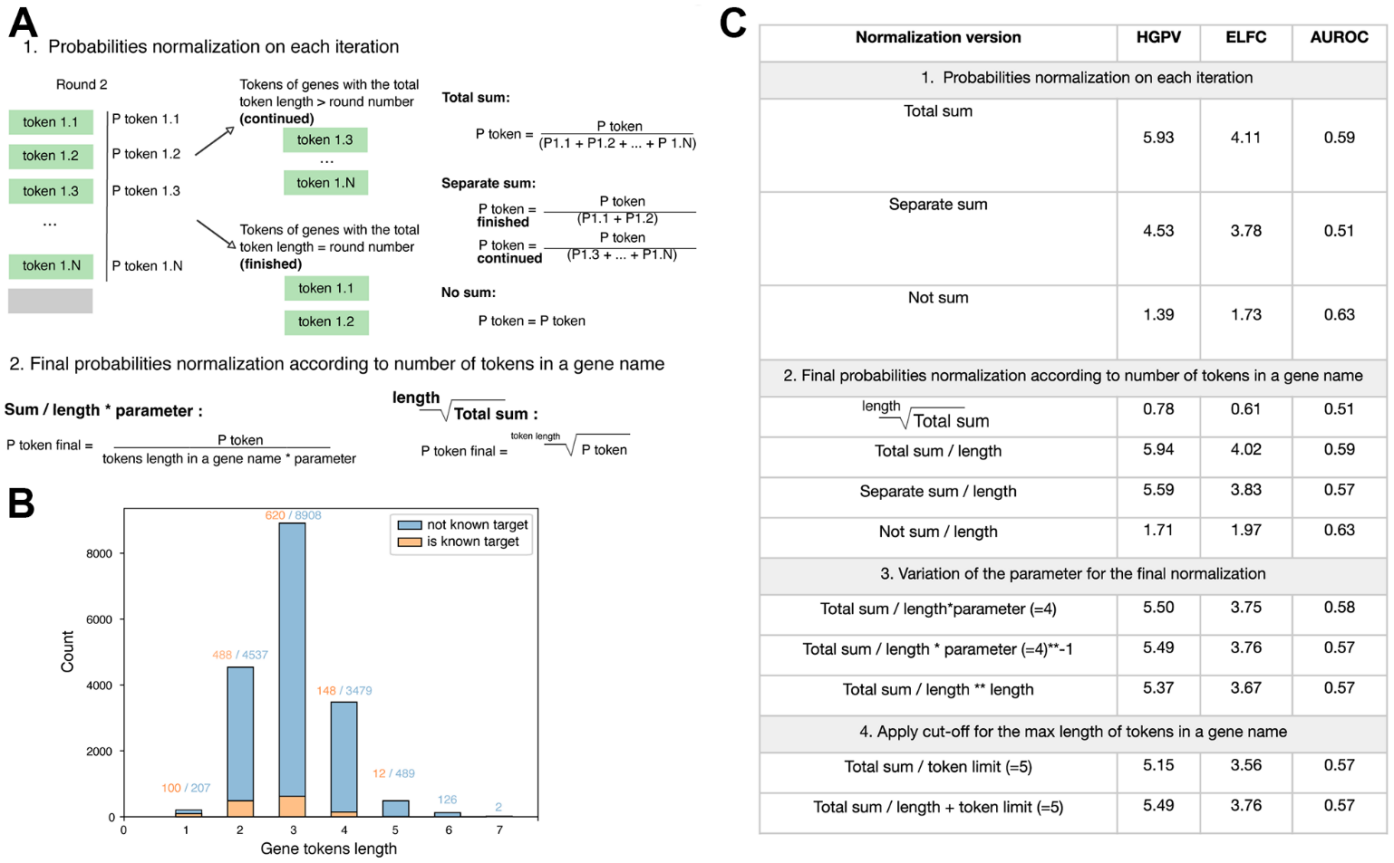
**Variations of the token probability normalization.** (**A**) Strategies for probability normalization at the step of the individual token probability retrieval (1) and final calculation of the total gene probability based on the tokens within its name (2). (**B**) Distribution of token lengths for protein-coding genes, for which the therapeutics are available (“is known target”) and not (“not known target”). (**C**) Validation metrics for the approaches of gene tokens normalization in the target identification task.

To account for the different token number in the gene name, we applied various normalization procedures and validated their effectiveness through internal testing. We identified two steps where the normalization could be applied, specifically, to the individual tokens on each iteration and the final product of the probabilities for all gene tokens. As only a small number of tokens corresponds to the gene names, we first tested individual tokens normalization at each step. Aiming to address the lowering of probabilities due to the prevalence of tokens not related to genes, we divided each token probability by the sum of probabilities of gene tokens (Figure 3A, “Total sum”). Also, we tested and considered the two approaches where genes finished in selected iteration (with the total token length equals to the round number) and continued genes (with the total token length exceeding the round number) separately (Figure 3A, “Separate sum”). According to the validation metrics, the normalization of the individual tokens in each iteration that resulted in the sum of all gene tokens equals to 1 has shown the best performance (Figure 3C). We next proceeded with the normalization of the final product of tokens probabilities. As the multiplication of several probabilities impaired the results for genes with longer names, we first applied approaches to correct for multiplication impact (Figure 3C, “Total sum ** 1 / length”). Additionally, we tested the approaches for decreasing the probability of genes with longer names, as we hypothesized that normalization procedure applied to each individual token could lead to overestimation of its value. Indeed, we observed the increase in metrics for the approach with the division of the final probability by the length of tokens within the gene name (Figure 3C, “2. Final probabilities normalization according to the number of tokens in a gene name”). Notably, the increase was observed in all tested combinations independent of the individual tokens normalization method. Additionally, we attempted to identify the best parameter for final normalization. The results suggested that parameter does not significantly affect the performance, however parameter equals to 1 yields slightly better results (Figure 3C, “3. Variation of the parameter for the final normalization”). Finally, we also observed that name lengths of known targets are distributed differently from all genes in total: only genes with names of 1 to 5 tokens are presented (Figure 3B). This observation is in line with the construction of the vocabulary for BioGPT that the most common genes, which are abundant in the training text datasets, appeared in the vocabulary as they frequently encountered. Thus, we also applied approaches for filtering out genes with the name length longer than 5 tokens. This cut-off was selected as no longer than this value was observed for known targets.

The results have shown that the model that involved the full list of genes, normalization to 1 on each interaction, and the normalization of the final product by the division of the name length outperforms other model versions. Altogether, we have shown that identification of word probabilities with LLMs should be carefully considered due to the limitation of the vocabulary size and identified the possible ways for normalization. Lastly, we have shown that the constructed pipeline allows efficient task-specific information extraction from LLM specifically used for the target discovery task.

### Training of BioGPT with the relevant to target discovery information increase the performance of the model

**Figure 4:**
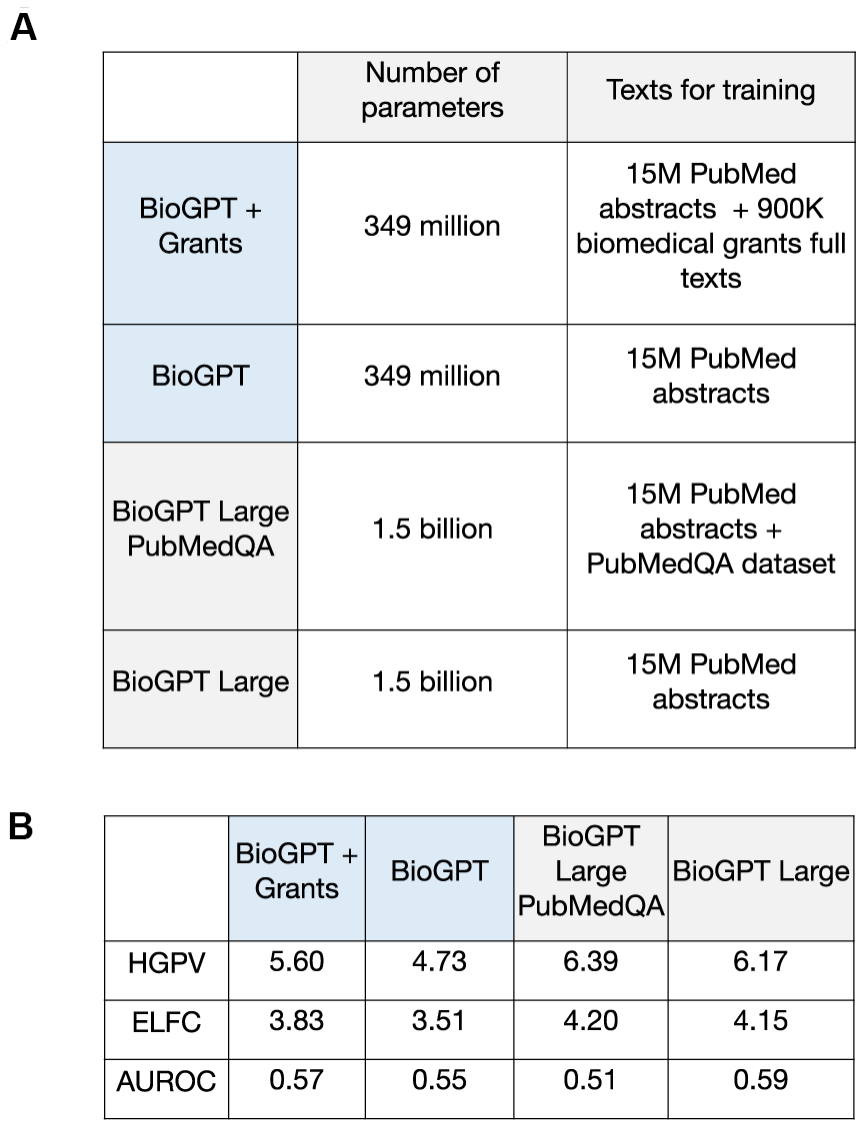
**Performance evaluation for BioGPT-based models in the target discovery task.** (**A**) The description of BioGPT versions considered for the evaluation in target discovery tasks. (**B**) Validation metrics for BioGPT models trained on different text corpus.

The established pipeline was used to estimate the performance of several modifications of BioGPT models to check if the training with additional data could improve the task-specific performance. Our main focus was on the initial BioGPT and the one trained with grants (BioGPT-G), but we also included two versions of BioGPT Large with increased parameter size: the basic one and the one trained with PubMedQA dataset (Figure 4A). Based on the validation metrics, training with additional relevant data promotes an increase in performance in target discovery tasks (Figure 4B). It was observed for both grants information and PubMedQA dataset. The general increase of BioGPT Large performance could be connected with the high parameters number.

Notably, the increase in performance was more pronounced for training with grants information compared with the PubMedQA. As PubMedQA dataset comprised only the short answers “yes/no/maybe” to biomedical questions that could be highly overlapped with PubMed abstract information [23], the grant abstracts could be more efficient for training and consequent model usage in target discovery. Altogether, pre-training of the established model with data that would be both different from the training set and relevant to the selected domain could be a powerful method for the task-specific model optimization.

### BioGPT-trained on grants is a powerful model in targets discovery

**Figure 5:**
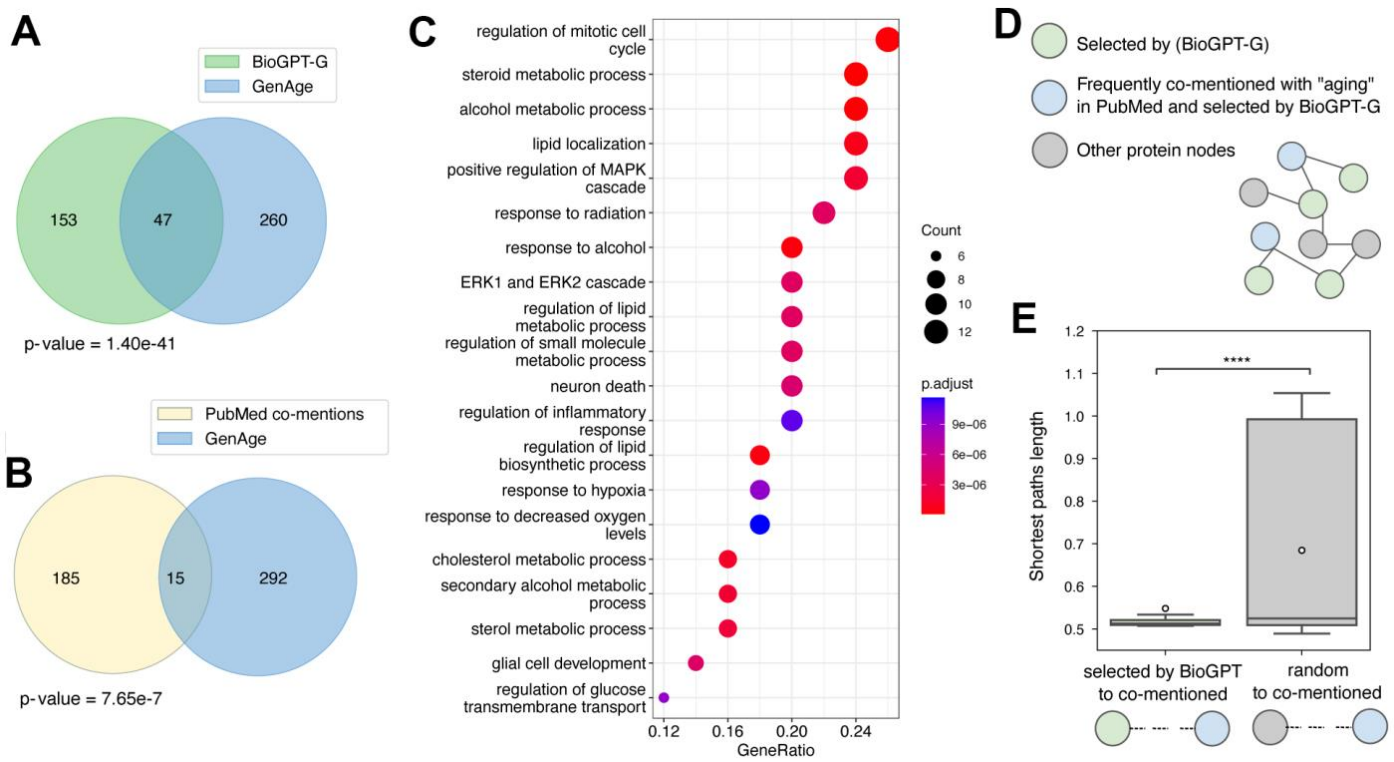
**Study of the top 200 age-related genes selected with the BioGPT-G model.** Venn diagram of the intersection of age-related genes obtained with an established approach based on BioGPT-G (**A**) or PubMed (**B**) and GenAge database data. Hypergeometric p-value is shown. (**C**) GO enrichment analysis for the top 50 genes ranked by BioGPT-G as age-related. (**D**) The proposed position of the graph nodes corresponding to the proteins appeared in different age-related lists. (**E**) Box plot of the shortest path length between the nodes of proteins selected by BioGPT or random nodes and the nodes corresponding to the proteins both selected by BioGPT and most frequently co-mentioned with “aging”. One iteration out of 1000 is shown for the random nodes. Asterisks indicate permutational test *p*-value: **** - *p* < 0.00001.

We next used a proposed approach for information retrieval with BioGPT-G to rank genes according to their association with aging. The top 200 entries (Supplementary Table 1) were selected for further investigation. Initially, we checked the intersection of this list with known age-related genes obtained from the GenAge database [24]. We have observed a significant overlap with 47 out of 200 genes appearing to have a known association with aging (*p* < 0.001; Figure 5A). For the comparison, we retrieved the genes that are most commonly mentioned in the context of aging in PubMed though calculating the number of abstracts with the co-occurrence of the selected gene name and “aging”. Their intersection with the GenAge database comprised 15 genes (*p* < 0.001; Figure 5B). Additionally, we also performed Gene Ontology (GO) enrichment analysis for the top 50 genes ranked by BioGPT-G and observed the significant results (FDR adjusted *p* < 0.01) for 575 categories (Figure 5C and Supplementary Table 2). Notably, most of the top ranked categories express the evidence for the association with aging. Specifically, cellular processes related to metabolism, stress response, and kinase cascades are described as age-related in recent publications [25–27]. Altogether, the results suggest that the usage of LLMs could be more efficient in context-specific tasks compared with the traditional target discovery methods.

We next aimed to gain more insights into the difference between the gene ranking with LLM and context search in PubMed. As standard ways of LLMs’ explainability methods are not established, we decided to focus on the investigation of protein embeddings. For that, we obtained protein representations with BioGPT and constructed the graph with vertex weights corresponding to cosine similarities between protein nodes. We hypothesized that during the training, BioGPT learns not only the probabilities of the words in the specific context, but also creates the internal associations of word similarities. Thus, we expected to see that nodes corresponding to the proteins, which are not highly mentioned with “aging” in PubMed texts but selected as age-related by BioGPT, should be placed closely on the graph to the proteins both co-mentioned with “aging” and selected as age-related by BioGPT (Figure 5D). As the measurement of the proximity, we selected the shortest path length. According to the results, the nodes of the proteins that were solely selected by BioGPT indeed lay significantly closer in the latent space compared with other nodes (Figure 5E). Thus, we proposed that along with efficient context-specific search the usage of LLMs allows leveraging more complex associations unavailable for other methods.

### Potential dual-purpose target discovery for aging and multiple age-related diseases

**Figure 6:**
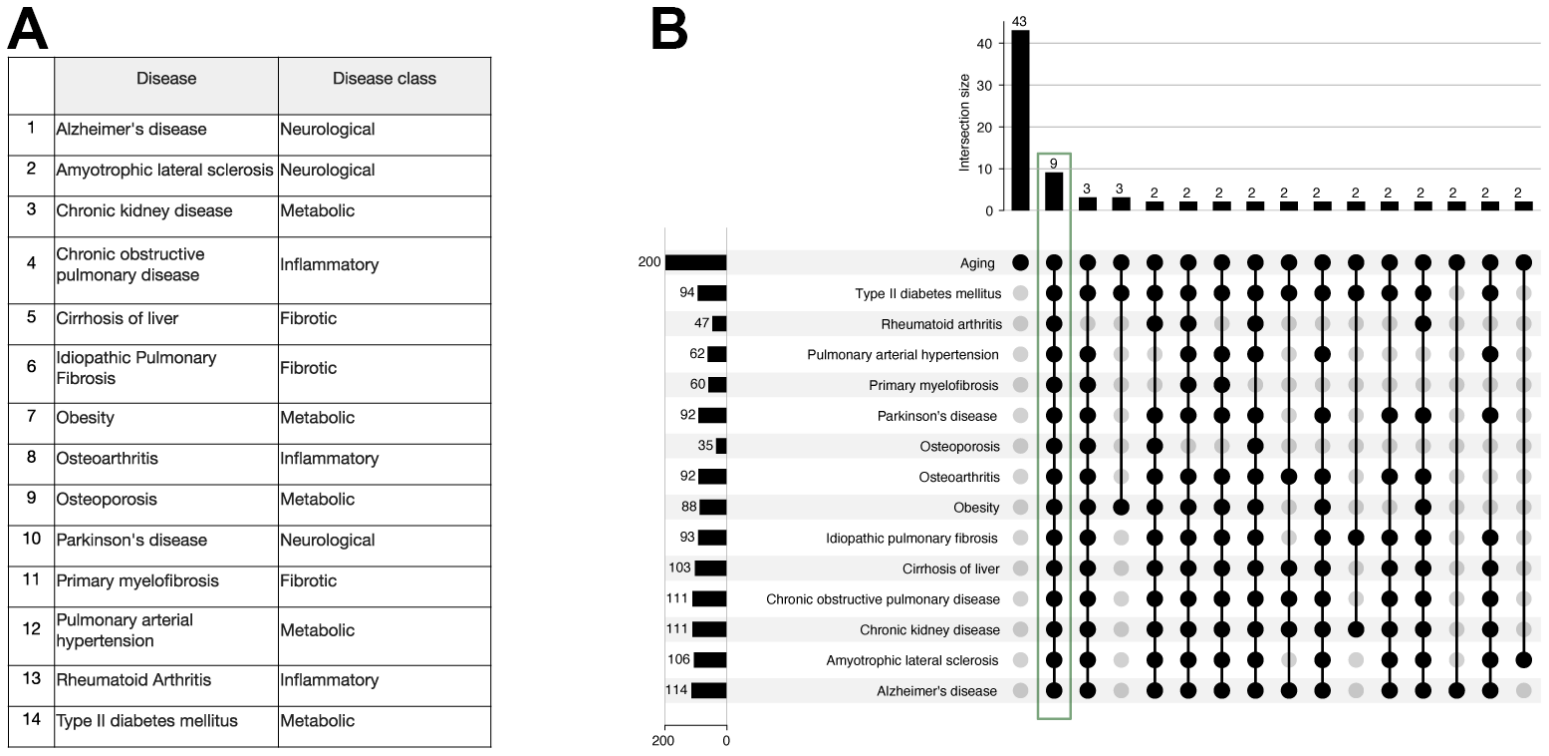
**Study of 9 genes that were ranked within the first 200 genes for each of the age-related diseases and aging by the BioGPT-G model.** (**A**) The list of 14 age-related diseases previously studied by Pun et al. (**B**) Upset plot of the intersection of protein lists for 14 diseases and aging retrieved with BioGPT-G. The intersection comprising 2 or more genes intersected with the “Aging” list are shown.

To proceed with the identification of the dual-purpose disease and age-related targets, we first retrieved the list of top 200 genes by BioGPT-G associated with each of the 14 age-related diseases (Supplementary Table 3), which were studied in one of the published works (Figure 6A) [28]. We then intersected the obtained lists with the results for the age-related genes search. According to the results, there were 9 genes common for all of 14 diseases and aging, specifically VHL, EGF, PTH, RET, BRCA1, SRC, CCR5, MIP, TNF (Figure 6B).

To investigate the novelty of the 9 obtained genes i.e., BRCA1, CCR5, EGF, MIP, PTH, RET, SRC, TNF, and VHL (Table 1), we have obtained additional data on the age-related targets including clinical trials and targets of drugs with anti-aging effect.

**Table 1:** A list of 9 targets predicted to be associated with aging and all 14 age-related diseases by BioGPT-G.

**Targets**	**Protein family**	**Clinical trial status^1^**	**Known as age-related genes^2^**	**Potential dual-purpose candidates^3^**
BRCA1	Acyltransferase	No	Yes	No
CCR5	GPCR	Yes	No	Yes
EGF	Growth factor	No	Yes	No
MIP	Generic protein	No	No	No
PTH	Generic protein	No	No	Yes
RET	Receptor kinase	Yes	Yes	Yes
SRC	Tyrosine kinase	Yes	Yes	Yes
TNF	Tumor necrosis factor	Yes	Yes	Yes
VHL	Ligase	No	Yes	No

^1^Any investigational or approved drugs associated with the targets.

^2^Genes were considered age-related if they were found on databases for experimentally validated age-related genes and compounds (DrugAge, GenAge, Geroproectors and Synergyage) and clinical trials data.

^3^Targets not considered as potential dual-purpose candidates were due to either (1) tumor suppressor properties or (2) opposite direction of therapeutic approaches between aging and age-related diseases.

Among these 9 genes, CCR5, MIP, and PTH are considered as novel age-related targets because their roles in aging were not reported previously based on the databases for experimentally validated age-related genes and compounds (DrugAge, GenAge, Geroproectors and Synergyage) as well as clinical trials data. Despite this, MIP is a candidate tumor suppressor, while BRCA1 and VHL are known tumor suppressors [29, 30]. The high scoring of tumor suppressors as associated with age and age-related diseases by our approach is in line with previous findings, which further suggested that tumor suppressor genes could be linked to aging [28]. However, targeting these tumor suppressors could be attributed to the increased risks of cancer development [31]. Thus, considering targets’ mechanisms of action according to the literature review, we propose three known age-related genes, TNF, SRC and RET, and two novel genes, CCR5 and PTH, as potential dual-purpose candidates for aging and age-related diseases.

Evidence suggested that TNF-α/IFN-γ synergy amplifies senescence-associated inflammation and that TNF-α antagonism can rescue the effect of aging on stroke [32, 33]. Moreover, dasatinib targeting SRC was considered as a senolytic used to remove senescent cells [34]. Furthermore, RET has been demonstrated to exhibit increased expression with age in rats [35]. RET binds to glial cell-line derived neurotrophic factor (GDNF) and other ligands to promote cell survival and proliferation via the PI3K/AKT and MAPK/ERK pathways [36]. In humans, RET is suggested to be associated with diverse subtypes of thyroid cancers [37]. Taken together, TNF, SRC and RET antagonisms are potential therapeutic approaches for aging and age-related diseases.

CCR5 is a co-receptor with CD4 for HIV infection, and the receptor for several inflammatory CC-chemokines, i.e., CCL3, CCL4, and CCL5, to subsequently activate AKT and NF-kB signaling pathways [38, 39]. It is predominantly expressed on the cell membrane of macrophages and T cells [40]. Given its close connection with chemokines, CCR5 coordinates immune cell differentiation and migrations, as well as promotes inflammation [41–43]. Pathologically, besides HIV, it has been implicated in cancer [44] and other inflammatory disorders, such as inflammatory bowel disease [45] and rheumatoid arthritis [46]. Blockade of CCR5 is suggested to be beneficial to treat these disorders [44, 47, 48]. Furthermore, CCR5 has been found to promote neuroinflammation, therefore inhibiting it can provide neuroprotective benefits [49]. Additionally, studies have shown that CCR5 can accelerate the development of Alzheimer's disease [50]. Cenicriviroc, which is a dual antagonist of CCR5 and CCR2, has been found to be effective in slowing the progression of non-alcoholic fatty liver disease [51] and reducing liver damage in rodents with cholestasis [52]. Therefore, CCR5 antagonism is a promising therapeutic approach for aging and age-related diseases.

Parathyroid hormone, encoded by *PTH* gene, maintains extracellular calcium and phosphorus homeostasis and facilitates renal vitamin D synthesis. PTH is synthesized in the parathyroid glands, and exerts its function in the kidneys, bones, and small intestine [53]. Mechanistically, upon the drop of extracellular calcium level, PTH stimulates calcium absorption in the small intestine and kidney, promotes renal phosphate excretion, and enhances bone resorption to increase calcium release in order to resume normal serum calcium level [54]. Serum levels of PTH were found to increase with age in humans [55] and are linked to age-related syndromes such as frailty [56], osteoporosis [57], and sarcopenia [58]. PTH administration in rats impairs energy production, transfer, and utilization in skeletal muscles [59], suggesting that PTH antagonism may be a potential therapeutic approach for aging and aging-related conditions.

In addition, considering their mechanisms of action and druggability, GDNF, NGF, BDNF, CXCR4, MYC and TH, being the top 200 aging targets that occur in more than 10 age-related diseases identified by BioGPT-G, were also considered as potential dual-purpose candidates.

## DISCUSSION

The advance in the natural language domain has permitted the development of LLMs, which have proven to exhibit remarkable performance in various complex tasks [60]. Despite the constant development of new models, the methods required for efficient information extraction from these models remains insufficiently understood. The lack of such approaches impedes the full potential of LLMs and significantly limits their applicability, especially in the domain-specific tasks. Therefore, our objective was to establish an approach for information retrieval from LLMs that can be applied to rank a given list of subjects. Specifically, we focused on the target prediction for the selected disease, which is among the most critical challenges in the biomedical field. We demonstrated that a ranked list could be generated from LLM through iterative calculation of the next token probability. Moreover, we have shown viable approaches to address the probability calculation for words containing more than one token. Our results demonstrated the high performance of the pipeline in target prediction, suggesting the potential of applying this method in the similar tasks of biomedical and other fields.

Among the various general language models available, there exist multiple pre-trained field-specific LLMs that are trained on in-domain data. One of the major sources of data for training biology-related LLMs is PubMed, which contains abstract and full texts of papers in Life Sciences [61]. We hypothesized that information used for training of domain-specific models may not be precise enough to allow the model to be efficiently used in narrow topics within this domain. Indeed, an analysis of the PubMed abstracts revealed that along with information on human health, this source contains articles related to other species and non-biological topics. Thus, we trained the BioGPT model [21], initially pre-trained by Microsoft on 15M PubMed abstracts, with an additional dataset containing texts relevant to target discovery. We assumed that research projects from National Institutes of Health (“grants”) could be useful for pre-training since biomedical grants descriptions mostly focus on drugs' impact on human health. Our results showed that training with grants could indeed enhance the performance of BioGPT in the target discovery task. Although training with grant texts was sufficient to show the model improvement, we suggest that larger and more comprehensive datasets or their combination could have a more significant impact on model performance. Also, we initially chose BioGPT for our work as a model with fewer parameters due to computational costs. We suggest that usage of BioGPT-Large trained on grants or other relevant data distinct from PubMed could additionally increase the performance in the target discovery task. Overall, we propose the potential benefits of an additional LLM model training on texts relevant to the selected task for improved downstream performance.

In this work, we focused on the application of the established pipeline to the identification of the potential targets related to aging, which is considered one of the most important risk factors for mortality. For that, we studied target prediction results for 14 age-related diseases and aging itself to identify the potential dual-purpose targets that are connected with both aging and multiple age-related diseases. We have also undertaken the task of providing a simple approach that could enhance the interpretability of BioGPT output by application of graph-based methods on nodes retrieved with protein embeddings. As explanation of LLMs behavior poses a known challenge [62], and only a limited number of approaches have been proposed, we suggest that investigating embeddings could serve as a straightforward yet promising approach towards a generalized explanation of LLMs result. Our initial findings indicate that the application of the pipeline yielded biologically meaningful results as some of the proposed genes were previously described in the literature as age-related or related to the specific disease, respectively. Moreover, the analysis of the genes most frequently selected both as related to aging and age-related disease by the model allows to identify the potential novel dual targets. Finally, we propose CCR5 and PTH as novel dual-purpose disease and age-related targets based on the comprehensive analysis including the evidence of participation in age-related cell pathways, druggability, known therapeutics mechanisms of action.

It is worth noting that our pipeline application is not limited to target selection. The ranking of a given list of subjects is a common biomedical task that involves revealing associations between biological terms such as genes, diseases, processes, drugs, etc. In addition to research purposes, this approach could assist medical doctors in conducting systematic reviews more efficiently by ranking relevant articles based on the selected topic. Furthermore, our pipeline can assist in ranking any subjects, even when a strict ranking criterion is not defined and a comprehensive understanding of large text corpuses is required. Therefore, we anticipate that our approach will have a wide range of potential applications in various areas, even beyond the biomedical field.

Despite the potential benefits of the proposed approach for target selection, there are several limitations to consider. As discussed in the paper, the comprehensive explainability of LLMs is still lacking. This makes it difficult to accurately assess the ability of LLM-based approaches to identify complex associations between genes and diseases. As a result, the identification of potential novel targets may be impeded, especially when there is restricted availability of various types of information such as scientific papers, grants, known therapeutics on the selected protein. Additionally, while the pipeline demonstrated high performance in target prediction, our validation was limited to only 14 age-related diseases. Therefore, it may be necessary to conduct more generalized performance estimation for a broader range of diseases and experimental validation of the predicted targets in order to obtain a more accurate estimation of the effectiveness of the proposed approach. Despite these limitations, our work demonstrates that pre-trained LLMs, integrated within a structured information extraction pipeline, can exhibit significant efficacy in performing complex tasks specific to a given domain. These results could provide a strong foundation for further investigations in this field.

## MATERIALS AND METHODS

### Data collection and disease selection

The list of genes was downloaded from The HUGO Gene Nomenclature Committee (HGNC) website [63] and filtered by the type. The final list of genes comprised 19,333 gene symbols and standard names. The genes most commonly co-mentioned with “aging” in PubMed abstracts were identified with BioPython [64]. The list of genes was ranked and top 200 genes by the co-mentions were selected. Also, the list of genes with the highest mention in the context of aging was retrieved. For that, we divided the number of publications where gene and “aging” were co-mentioned to the total number of publications mentioning the gene. This normalization was performed to account for the gene total prevalence within PubMed texts. Genes targeted by the investigated drugs that entered clinical trials with either aging or healthy aging as one of the disease conditions were obtained from the ClinicalTrials.gov. The list of genes that are targeted by compounds with anti-ageing properties were obtained by mapping of compounds found in DrugAge [65] and Geroprotector [66] databases to their known targets.

We downloaded around 900 thousand National Institutes of Health (https://report.nih.gov/) research projects, which obtained funding (“grants”). We proposed that funding for research is a good marker of valuable projects which contain valuable domain-specific knowledge. In this study we used abstracts of grants as texts for language model pretraining.

In addition, we obtained a random subset of 100 thousand PubMed (https://pubmed.ncbi.nlm.nih.gov/) article abstracts to analyze the common topics of this data source in comparison to grants topics.

For target identification in age-related diseases, a list of 14 previously published diseases according to the study of Pun et al. [28] was extracted. These diseases were selected based on their characteristics of having age as a strong risk factor for the disease’s onset.

### Text topic identification

Random sample of 100 thousand publications was selected from the PubMed abstracts and grant texts for the following analysis. First, embeddings were retrieved with “all-mpnet-base-v2” sentence-transformers model [67]. Next, texts were pre-filtered by the removal of the stopwords with NLTK (Natural Language Toolkit) [68] and conversion to lowercase format. Prepared PubMed and grant texts and their embeddings were used for the identification of the prevalent latent topics with BERTopic [22]. The minimum number of sentences per topic was set to 15. Identified dense clusters were visualized with the “visualize_documents” function.

### BioGPT-G training

We initially took a BioGPT model with 347 million parameters which is based on GPT-2 [69] medium size and pre-trained on 15 million PubMed abstracts. The training objective of this model is standard language modeling task, which aims to maximize log-likelihood of a next token given the context (def 1):


$$def\;1\;:\;maximize\;\sum_{t=1}^{M}log_{2}p(y_{t}\;\vee y_{t}),$$

where $p(y_{t}\;\vee y_{t})$ is a conditional probability, *M* is a number of words in a context.

**Figure 7:**
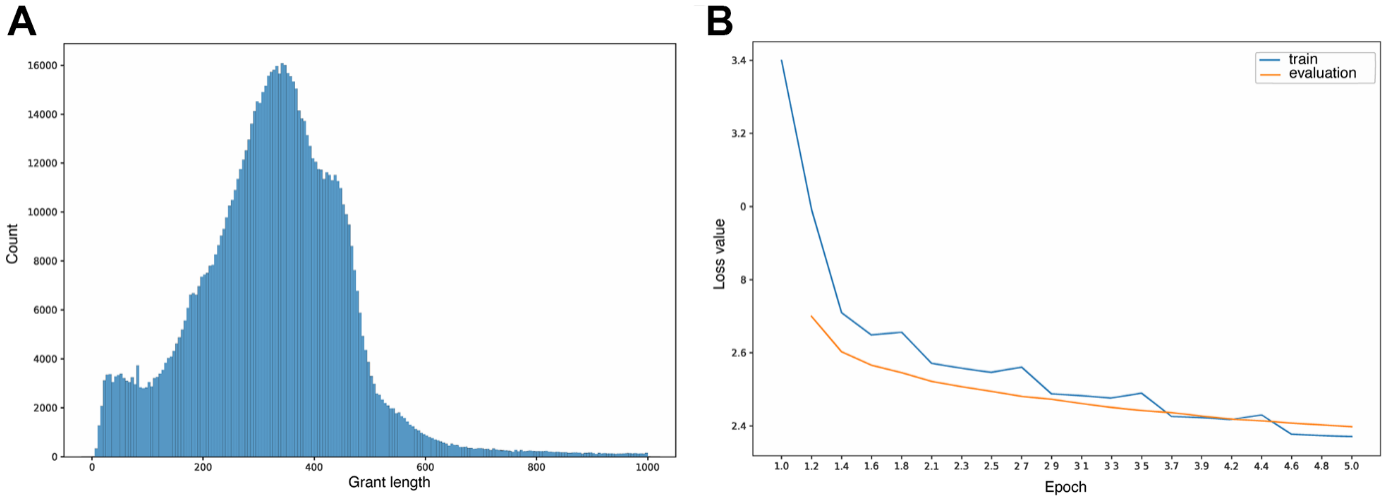
(**A**) Learning curves. (**B**) Grant lengths distribution.

To enrich the biomedical knowledge base of the chosen BioGPT model, we additionally pretrained it on more than 900 thousand grant abstracts by optimizing the same objective as for GPT-2 [69]. The pre-training lasted for around 40 hours with a train batch size of 16 and gradient accumulation steps of 64 per device on four A5000 GPUs. For the optimization, we used Adam algorithm [70] with 100 warm-up steps and learning rate 5e-5. The original BioGPT tokenizer was used with the max length of input texts equal to 250 tokens with truncation, the distribution of input text lengths depicted in Figure 7A. We filtered out abstracts with less than 8 words total assuming that they don’t carry useful information.

The training procedure was defined to 5 epochs due to time and computational resources limits, which is not enough for full model convergence, but still close to training and evaluation losses plato (Figure 7B).

### Model validation

The sorted list of targets was produced for each model type and the top-k validation method was implemented/ The top-k gene list was evaluated on the effect strength and effect significance based on the log fold change of enrichment (ELFC) and hypergeometric *p*-value (HGPV) scores respectively. ELFC estimates whether the percentage of source nodes for a particular target node has increased within the top-k relative to the total percentage of known source nodes associated with this target node. In the application to the current task, the effect strength estimates the percentage of ADRs that were scored in top-k to a specific drug and, similarly, the percentage of drugs that were scored in top-k to a specific protein. ELFC was calculated by the following formula:


$$ELFC(score)=log2\left(\frac{targets_{k}*N}{k*targets_{N}}\right),$$

where targets_k_ was the number of known targets for this disease in top-k (or 0.1 if there were none), and targets_N_ was the total number of known targets for this disease.

Along with the effect strength, its significance was estimated based on the top-k hypergeometric test. HGPV score was calculated based on the following formula:


$$HGPV(score)=-log10\left(1-hgcdf(targets_{k},k,targets_{N},N)\right),$$

where hgcdf is a hypergeometric cumulative distribution function. *P*-values indicate the probability of drawing targets-k or more known targets in k draws from a set of N genes with targets-N known targets. Thus, HGPV score represents -log10 of such *p*-values for each target node. Higher values of ELFC and HGPV corresponded to the higher predictive power of the protein-ADR association.

### Protein representation retrieval and graph construction

To get proteins representations, the input text containing the prompt “The human age-associated gene is the” and the name of one of the genes was first tokenized and converted to PyTorch tensor [71] Then, token embeddings were computed with the following mean output pooling. The resulting vectors with the length of 1024 corresponding to the individual proteins were used for the analysis. The similarity between the proteins were represented as cosine similarities between the embeddings, which were further used as vertices for the weighted graph.

The weighted undirected graphs were constructed with NetworkX software [72]. First, the links between the proteins were filtered based on the cosine similarity cut-off equals to 0.507 or the upper quartile of the cosine similarities distribution. Then, the shortest path was calculated using Dijkstra’s algorithm. As the source nodes the nodes corresponding to the proteins both co-mentioned in PubMed abstracts with “aging” and selected as associated with aging by BioGPT. For the targets, the nodes corresponding to the proteins selected as associated with aging by BioGPT but not co-mentioned in PubMed abstracts and random proteins nodes for the main experiment and control were selected, respectively.

## Supplementary Material

Supplementary Table 1

Supplementary Table 2

Supplementary Table 3
